# Three critical factors affecting automated image species recognition performance for camera traps

**DOI:** 10.1002/ece3.6147

**Published:** 2020-03-07

**Authors:** Stefan Schneider, Saul Greenberg, Graham W. Taylor, Stefan C. Kremer

**Affiliations:** ^1^ School of Computer Science University of Guelph Guelph ON Canada; ^2^ Department of Computer Science University of Calgary Calgary AB Canada; ^3^ School of Engineering Vector Institute of Artificial Intelligence University of Guelph Guelph ON Canada

**Keywords:** camera traps, computer vision, convolutional networks, deep learning, density estimation, monitoring, population dynamics, species classification

## Abstract

Ecological camera traps are increasingly used by wildlife biologists to unobtrusively monitor an ecosystems animal population. However, manual inspection of the images produced is expensive, laborious, and time‐consuming. The success of deep learning systems using camera trap images has been previously explored in preliminary stages. These studies, however, are lacking in their practicality. They are primarily focused on extremely large datasets, often millions of images, and there is little to no focus on performance when tasked with species identification in new locations not seen during training. Our goal was to test the capabilities of deep learning systems trained on camera trap images using modestly sized training data, compare performance when considering unseen background locations, and quantify the gradient of lower bound performance to provide a guideline of data requirements in correspondence to performance expectations. We use a dataset provided by Parks Canada containing 47,279 images collected from 36 unique geographic locations across multiple environments. Images represent 55 animal species and human activity with high‐class imbalance. We trained, tested, and compared the capabilities of six deep learning computer vision networks using transfer learning and image augmentation: DenseNet201, Inception‐ResNet‐V3, InceptionV3, NASNetMobile, MobileNetV2, and Xception. We compare overall performance on “trained” locations where DenseNet201 performed best with 95.6% top‐1 accuracy showing promise for deep learning methods for smaller scale research efforts. Using trained locations, classifications with <500 images had low and highly variable recall of 0.750 ± 0.329, while classifications with over 1,000 images had a high and stable recall of 0.971 ± 0.0137. Models tasked with classifying species from untrained locations were less accurate, with DenseNet201 performing best with 68.7% top‐1 accuracy. Finally, we provide an open repository where ecologists can insert their image data to train and test custom species detection models for their desired ecological domain.

## Graphical abstract

Here, we present the capabilities of deep learning computer vision system for species identification from camera trap images. We compare when background locations are found within the training data, while also excluded. In addition, we also provide guidelines for the number of images required for ecologists when considering their ecosystem of interest.

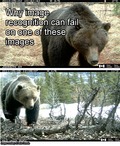

## 1 INTRODUCTION

Camera traps are cameras set up at strategic field locations. They can be configured to take periodic images over time or to respond to motion such as an animal entering the field of view. Wildlife ecologists use camera traps to monitor animal population sizes and manage ecosystems around the world (O'Connell, Nichols, & Karanth, 2010). Camera traps were first introduced in 1956, and in 1995, Karanth demonstrated their usefulness for population ecology by reidentifying tigers (*Panthera tigris*) in Nagarahole, India, using a formal mark and recapture model (Gysel & Davis, 1956; Karanth, 1995). The popularity of the camera trap methodology grew rapidly thereafter, with a 50% annual growth using the technique as a tool to estimate population sizes (Burton et al., 2015; Rowcliffe & Carbone, 2008).

Projects involving camera traps can accumulate thousands to millions of images and provide a rich source of data. The problem is that camera trap data analysis requires a person to manually inspect each image and record its attributes, such as quantifying the species and number of individuals seen in an image. Automating this process has obvious advantages, including a reduction in human labor, an unbiased estimate across analyses, and the availability of species identification without domain expertise.

To solve this problem, many researchers are exploring deep learning computer vision models as a powerful tool, where image recognition techniques are used to detect and/or classify ecological entities (such as wildlife) seen in an image. Ideally, high classification accuracy will mitigate the ecologist's laborious task of extracting ecological information from camera trap images. Recent results are encouraging, where some report species recognition accuracy up to 98% in certain conditions (Tabak et al., 2019; Willi et al., 2019). Yet, many of these models also make assumptions about data availability that limit their applicability to all ecological practice.

Our work investigates how recognition accuracy is affected by various factors, as outlined in the next section. In particular, we want to understand—and quantify where possible—the boundary conditions of each factor, that is, the transition between a deep learning method performing well versus poorly/fails to perform. Performing well implies a model returns consistent, high accuracies/recall, while performing poorly/fails to perform implies either highly variable prediction accuracies/recall and/or an overall inability to make accurate predictions. By understanding the capabilities and limitations of deep learning systems, ecologists can then use this knowledge as indicators of whether a technique is useful for their particular circumstance. This includes the classification accuracy/recall they can expect, how much additional effort would be required to expect a well‐performing model, and indicators of when the model is under performing.

### 1.1 Challenges

In this work, we focus on deep learning computer vision approaches for ecological image classification catered to an ecological audience. Our focus is threefold: How well do several deep learning models perform in terms of classification accuracy when trained on a modest modestly sized labeled dataset; how well do deep learning models generalize to images taken at locations not seen during training; and how is classification accuracy per species affected by the amount of training data for that species (especially when training data are limited). These questions have been touched upon by a handful of others, but even so were not considered in depth. For example, that prior work uses very large training sets comprising millions of images and did not consider the gradient/lower bound for which deep learning methods fail to perform in terms of data limitations (Tabak et al., 2019). We explore the challenges associated with deep learning for practical application for smaller scale ecological research efforts, where our results can guide ecologists in understanding where the technology may be applicable to one's work, and what levels of performance one should expect in various conditions.

Deep learning on ecological image data introduces multiple challenges of which, if not taken into account, can affect its practical application. These challenges include the following:

#### 1.1.1 Size of the training set

Many deep learning researchers train models using a dataset containing a massive number of labeled images (Norouzzadeh et al., 2018; Tabak et al., 2019; Willi et al., 2019). However, such large training numbers are likely impractical for the vast majority of smaller scale ecological research projects, as labeled data particular to a project must come from domain experts and are thus limited.

#### 1.1.2 Application to new locations

*Domain shift* occurs when images used for training are taken from a field that differs from those used for testing (Csurka, 2017). Deep learning systems perform best under conditions where the training and testing environments are as similar as possible (Goodfellow, Bengio, & Courville, 2016; LeCun, Bengio, & Hinton, 2015). Indeed, many tests of deep learning systems embody this assumption. However, training models on camera trap images are particularly susceptible to this challenge due to the static nature of the background training images (Beery, Van Horn, MacAodha, & Perona, 2019; Howard, 2013; Krizhevsky, Sutskever, & Hinton, 2012). This means that accuracies reported on the majority of deep learning camera trap papers only reflect locations seen during training and, especially when considering limited data, will underperform in new locations (Tabak et al., 2019). Practically, ecologists will often want to classify images from a camera situated at a new location whose images were not included in the training set (Meek, Vernes, & Falzon, 2013). Those images should be considered as a different domain as they can differ considerably from the trained images, for example, different backgrounds (grasslands, forest, etc.), different prominent objects (tree stumps, logs, rocks), and different environmental conditions (lighting, shadow casting, summer vs. winter). We argue—and will show—that classification accuracy for camera images taken at such untrained locations can differ considerably from images taken from trained locations and should be considered as a standardized metric (Tabak et al., 2019). How well a model responds to new domains is known in the deep learning field as *generalization* (Goodfellow et al., 2016).

#### 1.1.3 Imbalanced datasets

Datasets are often imbalanced. While some species are frequently represented across many images, images of other species are sparse (Chao, 1989; Krizhevsky et al., 2012). Imbalance can negatively affect classification accuracy for a poorly represented species, as the deep learning model may not have seen sufficient training images of that species (LeCun et al., 2015). For example, while the model's accuracy, recall, and precision may be high across all species, performance for rarely seen species may be considerably lower. A few previous works have identified this issue, but have not demonstrated the degradation in performance as data decreases <1,000 images per species of interest. We explore that here.

### 1.2 Research goals

Our research goal is motivated by the above challenges. To test the limits of image recognition capabilities, we consider the performance of six deep learning systems on an ecological domain, as represented by a modest‐sized dataset that could be practically collected by smaller research groups, unbalanced in how species are distributed across images, and *messy*, that is, with animals being partly obstructed, positioned at varying distances, cropped out of the image, or extremely close to the camera (Norouzzadeh et al., 2018). We report model performance under two conditions: when test locations are seen during training and when they are not. We realize this goal in part by using a labeled dataset offered by Parks Canada—Canada's largest environmental agency—which embodies characteristics typical of ecological data (Imgaug github repository‐b). Our subgoals are to answer the following questions to provide guidelines for ecologists.

#### 1.2.1 Quantify the performance of a range of deep learning classifiers given a modest camera trap dataset

We want to understand how a modest‐sized training set affects image classification accuracy. This can help an ecologist determine a cost/benefit threshold, where a modest amount of image training can produce a reasonable accuracy level. In particular, we limit our model training and accuracy testing to the 47,279 images provided in the Parks Canada dataset, with some exceptions as described in the Section 3. These images are taken “as is” and include the many messy attributes as previously described. We perform our tests using several different deep learning classifiers to see how well each one performs, and how they perform collectively.

#### 1.2.2 Quantify generalization to new untrained locations

We want to test whether a model's classification accuracy differs between images taken from trained locations versus images taken from untrained locations, across a sparse number (36 in our case) of unique geographic locations of varying environments by measuring performance on trained and untrained locations. Untrained locations mimic expected camera trap usage, as biologists often deploy cameras to new locations over time. We test this using a k‐fold validation split.

#### 1.2.3 Quantify the gradient of class‐specific performance as data increases

For each species, we count the number of images available during training and report the recall of the model output. We then correlate this using a logarithmic regression across all species to determine a threshold of the number of images required per species to achieve reasonable recognition accuracy. We also document the sporadic and unreliable behavior of deep learning models when training data for a particular class are limited.

Our results are promising with caveats. To summarize, we find that high overall classification accuracy, >95%, can be achieved even with limited‐sized datasets when making predictions on novel classifications with 1,000+ training images taken from locations seen during training. This is promising for ecological research efforts that do not relocate their camera traps. We also find that classification accuracy degrades with untrained locations. That is, if one trains a model using camera trap images captured from a small number of geographic locations (and thus a small number of image backgrounds) and then tries to classify images from a camera trap at a new location (and thus different background), accuracy decreases significantly to approximately 70% (Tabak et al., 2019). We note that Tabak et al. (2019) also identified this issue but with a smaller decrease in recognition accuracy, which we ascribe to them using millions of training images (and thus a larger number of backgrounds) and only testing on a single untrained location. In contrast, we wanted a more robust measure of a model's generalization using a modest‐sized training set to many novel locations. As will be discussed later, we argue that studies of deep learning classification tasks for camera traps should be standardized to include many novel locations by using k‐fold validation (see our Section 3). Lastly, while previous experiments have demonstrated small degradation dips in performance relative to thousands of training images per species, we document—for a given species—the highly variable, unreliable behavior of machine learning models when less training data are available for that species (Tabak et al., 2019). For example, we approximate—for trained locations—that 500, 750, and 1,000 training images per species are will achieve recalls above 0.750, 0.874, and 0.971, respectively, for that species considering our camera trap dataset. These results provide finer granularity about what happens when training data have fewer images when compared to previous works (Tabak et al., 2019) and can serve as rough metrics for ecologists considering deep learning for their ecological camera trap task.

While we quantify our results, our exact numbers should be taken as a rough estimate of what to expect in other situations. Our results are based on a single dataset, and our experiment should be replicated on other datasets. To encourage replication, we make our code and training/testing pipeline (written in Python) publicly available for other ecological groups to train their own models and to generate their own results and to compare results as a community (Schneider github animal classification tool).

## 2 BACKGROUND AND RELATED WORK

### 2.1 A general overview of deep learning for image classification

Prior to the widespread adoption of deep learning systems, computer vision researchers developed a variety of creative and moderately successful methodologies for the automated analysis of animals seen in camera trap images based on the raw pixel data from images. Initial approaches for species classification required a domain expert to identify meaningful features for the desired classification (such as the defining characteristics of animal species), design a unique algorithm to extract these features from the image, and compare individual differences using statistical analysis. Computer vision systems were first introduced for species classification within the microbial and zooplankton community to help standardize species classification and zooplankton morphology considering their silhouettes (Balfoort et al., 1992; Jeffries et al., 1984; Simpson, Culverhouse, Ellis, & Williams, 1991). From 1990 to 2016, species identification from camera traps focused on feature extraction methods. After 2016, the focus turned to using deep learning for species classification (Schneider, Taylor, Linquist, & Kremer, 2019).

Deep learning has seen a rapid growth of interest in many domains, due to improved computational power and the availability of large datasets (LeCun et al., 2015; Schneider, Taylor, & Kremer, 2018; Schneider et al., 2019). The term *deep learning* describes the use of a statistical model, known as a neural network, containing multiple layers to solve the problem of data representation. The statistical model is created via *training*, where the model is built from a (typically large) set of inputs and known labeled outputs (LeCun et al., 2015). Neural networks are composed of a series of layered nonlinear transformations using modifiable parameters/weights that update relative to the training data seen (LeCun et al., 2015). This statistical structure allows for mapping of logical relationships from input data to output classification if a relationship exists (Hornik, 1991). In recent years, deep learning methods have dramatically improved performance levels in the fields of speech recognition, object recognition/detection, drug discovery, genomics, and other areas (Amodei et al., 2016; Eraslan, Avsec, Gagneur, & Theis, 2019; He, Gkioxari, Dollár, & Girshick, 2017).

In the case of ecological camera trap images, the input sources are an image's RGB (red, blue, and green) pixel channels, and the output is species class. However, the model must first be trained, typically by providing the deep learning system with a large set of labeled images that have previously been classified by the analyst. The deep learning system can then compare subsequent unlabeled images against this model and determine the classification label that best fits it. The model's classification outputs are typically reported as a set of per‐class probabilities.

Many recent advances in deep learning come from customizing the layers for specific classification tasks, such as for images. One such layer is the “convolutional layer” used in convolutional neural networks (CNNs), which are now the most commonly used network for computer vision tasks (Fukushima, 1979; Krizhevsky et al., 2012). Convolutional layers learn *feature maps* representing the spatial similarity of patterns found within the image, such as color clusters, or the presence or absence of lines (LeCun et al., 2015). CNNs also introduce *max‐pooling layers*, a method that reduces computation and increases robustness by evenly dividing these feature maps into regions and returning only their maximum value (LeCun et al., 2015). The pattern of these layers comprises what is known as a networks *architecture*. Many networks architectures have standardized due to their landmark performance and are readily publicly available for commercial and scientific purposes. Such network architectures include the following: AlexNet (the first breakthrough CNN), VGG19 (a well‐performing 19 layered CNN), GoogLeNet/InceptionNet (which introduced the inception layer), and ResNet (which introduced residual layers) among many others (He, Zhang, Ren, & Sun, 2016; Jaderberg, Simonyan, & Zisserman, 2015; Krizhevsky et al., 2012; Szegedy et al., 2015).

Deep learning researchers continually experiment with the modular architectures of neural networks, generally at the trade‐off between computational cost and memory to accuracy. For our experiment, the models we chose appear on a gradient of increasing complexity: MobileNetV2, NASNetMobile, DenseNet201, Xception, InceptionV3, and Inception‐ResNet‐V2. Architectures like MobileNetV2, which is 14MB in size, are catered to low computational overhead in lieu of the ability to map complex representations. In contrast, Inception‐ResNet‐V3, which is 215MB in size, requires high computational overhead but maximizes representational complexity (Redmon, Divvala, Girshick, & Farhadi, 2016). In practical terms, understanding the relative accuracy of these models on ecological images versus their computational complexity will help map out the classification benefit versus the computational cost of choosing a particular model.

A bottleneck to classification accuracy is the number of labeled images available for training, as the model must be trained on many images in order to produce accurate classifications. A common approach to training deep learning classifiers on limited datasets, such as ecological camera trap images, is to perform *image augmentation*. Image augmentation refers to the introduction of variation to an image, such as mirroring, shifting, rotation, blurring, color distortion, random cropping, nearest neighbor pixel swapping, among many others (Imgaug github repository‐a). This approach creates new training images, which allows a computer vision network to train on orders of magnitude more examples that uniquely correspond to the provided labeled output classifications. This is a desirable alternative due to the expensive cost (or unavailability) of collecting and labeling additional images. A second common approach to improve training on limited data is *transfer learning*, where one initializes the weights of a standardized network using their publicly available weights trained on a large dataset, such as ImageNet (Krizhevsky et al., 2012). This allows for learned filters, such as edge or color detectors, to be used by the model for a particular niche domain, without having to be relearned on limited data (Pan & Yang, 2010). Both these techniques are used within our work, and in our provided public repository, as they can help improve the model's accuracy at little extra cost.

In summary, deep learning methods are developing as a promising method for automatically classifying ecological camera trap images. Yet, practical problems exist as listed earlier that can affect classification accuracy: Labeled datasets used for training may be limited, ecological images are messy and imbalanced, and images to be tested may be domain‐shifted when they come from camera locations not seen during training. Consequently, we need to better understand the capabilities of deep learning systems on an ecological domain, especially in context of smaller projects, as is detailed in the remainder of this paper.

### 2.2 Previous deep learning methods applied to camera trap images

In 2014, Chen, Han, He, Kays, and Forrester (2014) authored the first paper for animal species classification using a CNN that considered the Reconyx Camera Trap dataset. Their CNN was a shallow network by modern standards, with three convolution and three pooling layers. Considering approximately 23,876 images from 20 classes of North American species, their model returned 33.5% accuracy. In 2016, Gomez, Salazar, and Vargas (2016) used deep CNNs for camera trap species recognition, comparing eight variations of the established CNN frameworks AlexNet, VGG, GoogLeNet, and ResNet to train species classification on the complete Snapshot Serengeti dataset of 3.2 million images with 48 classes of species. The ResNet‐101 architecture achieved the best performance with 88.9% accuracy. Following this work, they also used deep learning to improve low‐resolution animal species recognition by training deep CNNs on poor‐quality images. The data were labeled by experts into two datasets, the first classifying between birds and mammals and the second classification of different mammal species (Caruana, 1998; Gomez, Diez, Salazar, & Diaz, 2016).

In 2018, Norouzzadeh et al. (2018) explored a network's ability to yield several distinct predictions for a given image (known as multitask learning) to classify the species, quantify the number of animals, and to determine additional attributes. This approach operates differently than single classification detection methods, as their classifier is trained to output multiple predictions, including species, number of individuals, and youth versus adult. Nine independent architectures were trained, including AlexNet, VGG, GoogLeNet, and numerous variations of ResNet. The authors report a species classification and counting accuracy of 94.9% and 63.1%, respectively (Norouzzadeh et al., 2018). In 2019, Tabak et al. trained the ResNet‐18 architecture on the 3.7 million images contained within the North American Camera Trap Images dataset, testing four ungulate species on a single unseen location, which returned 81.8% accuracy (Tabak et al., 2019). Willi et al. (2019) expanded on this, by showing the success of citizen science programs for deep learning and by successfully training classifiers of high performance considering four datasets of >400,000 images. Schneider et al. (2018) demonstrated and compared the capabilities of the Faster R‐CNN and YOLO V2 object detection methods and how they quantify and localize animal species from two different camera trap datasets, the Gold Standard Snapshot Serengeti and the Reconyx camera trap dataset. Beery, VanHorn et al. (2019)) started an annual camera trap data science competition focused specifically on methods improving generalization: Any individual can participate and view their results. In 2019, Beery, Wu, Rathod, Votel, and Huang (2019) recognize the utility of static backgrounds from camera trap images and introduce a unique network architecture catered to this scenario. Several large technology firms also showed philanthropic support for automated classification of camera trap images. In 2019, Google subsidiary company DeepMind (a renowned leader in artificial intelligence) announced a global initiative to use machine learning to accelerate ecological research (Using machine learning to accelerate ecological research). Somewhat similarly, the goal of Microsoft's AI for Earth is to put “AI tools in the hands of those working to solve global environmental challenges,” where a subproject includes deep learning for detecting and classifying wildlife in camera trap images (Ai for earth).

## 3 MATERIALS AND METHOD

### 3.1 The Parks Canada dataset

The dataset used in our experiment was provided by Parks Canada, Canada's largest government‐funded environmental agency. This agency represents thousands of terrestrial and marine conservation areas, and employs approximately 4,000 conservationists (Imgaug github repository‐b). Parks Canada has a rich history of ecological monitoring and is currently exploring the application of deep learning to extract ecological information from camera trap data (Imgaug github repository‐b).

The Parks Canada dataset was provided by one of their field units. It is a real in‐the‐field dataset. Images are from field cameras deployed at strategic locations parks across in Alberta, Canada, as determined by their staff and manually labeled by resource conservation officers as part of their own ongoing conservation work. They provided images from 36 camera trap locations, each a unique geographic location spanning a variety of environments, including dense forest, riverways, mountain cliff sides, and open plains. While Parks was interested in seeing the potential of automatic classification, the dataset was collected and provided to us without them having any knowledge of the experimental details. As such, their dataset represents a unique scenario for exploring the success of deep learning models considering real world but limited data. Use of these data required a confidentiality agreement, as their privacy policy disallows public dissemination of camera trap images that contain people.

The dataset consists of 47,279 images and considers 55 classes of animal species or human activity without animals (e.g., hiker, hunter) across 36 locations from Alberta's parks. Camera trap images are captured as a motion‐triggered sequence of five images taken 1 s apart and labeled with the corresponding correct classification by a Parks Canada member, as well as a thorough second pass by us. As common with many ecological datasets, images are messy: They capture a variety of day/night lighting conditions and seasonal weather, and animals at various distances, poses, and partially obstructed or cropped out of the image. The dataset also includes a considerable number of images where no animal is present (9,039 images). The dataset was also class imbalanced. This is dramatically illustrated in Table 1, which orders the frequency that a species is represented in an image. As seen in that table, there are thousands of images of some species (such as 7,904 white‐tailed deer and 5,735 elk) but only a much smaller number of images of other species (such as 108 porcupines, 79 badgers, and only 10 woodrats).

**Table 1:** Training testing split and recall per species for trained locations

Species	Num training images	Num testing images	DenseNet 201	Inception‐ResNet‐V2	Inception V3	MobileNet V2	Xception	NASNet Mobile	Ensemble
No Animal	8,566	473	0.966	0.947	0.962	0.926	0.962	0.943	0.970
White‐tailed Deer	7,484	420	0.990	0.962	0.969	0.968	0.963	0.972	0.976
Elk	5,426	309	0.987	0.968	0.974	0.977	0.977	0.974	0.981
Wolf	5,269	246	0.955	0.939	0.935	0.967	0.963	0.971	0.967
Grizzly Bear	3,803	211	0.986	0.976	0.962	0.981	0.986	0.986	0.995
Mule Deer	2,785	158	0.962	0.949	0.918	0.930	0.956	0.949	0.968
Snowshoe Hare	2,328	110	0.955	0.918	0.945	0.864	0.964	0.800	0.936
Bighorn Sheep	1,050	57	0.965	1.0	0.982	1.0	1.0	1.0	1.0
Coyote	893	53	0.774	0.925	0.925	0.925	0.943	0.811	0.943
Lynx	854	45	0.956	0.956	0.956	0.978	0.933	0.844	0.956
Black Bear	761	34	0.971	0.853	0.941	0.912	0.941	0.529	0.971
Red Fox	688	36	0.750	0.778	0.694	0.722	0.861	0.778	0.806
Cougar	635	29	1.0	0.333	1.0	0.667	1.0	1.0	1.0
Moose	512	24	0.916	1.0	0.792	0.917	0.958	0.583	0.958
Canada Goose	461	31	1.0	1.0	1.0	1.0	1.0	1.0	1.0
Marten	223	10	0.600	0.700	0.700	0.700	0.900	0.700	0.700
Vehicle	199	8	1.0	0.750	1.0	0.875	1.0	1.0	1.0
Wolverine	187	11	0.812	0.722	0.812	0.812	0.812	0.636	0.812
Horse Rider	159	10	0.800	0.700	0.400	0.800	0.800	0.800	0.800
Hiker	153	8	1.0	0.375	0.750	0.875	0.750	0.625	1.0
Snowmobile	146	9	1.0	1.0	0.889	0.889	1.0	0.667	0.889
Dog	135	7	0.571	0.571	0.857	0.286	0.571	0.429	0.714
Horse	122	8	0.875	0.750	1.0	1.0	1.0	0.875	1.0
Mountain Goat	119	8	1.0	1.0	0.875	0.750	0.875	0.750	1.0
Bobcat	106	2	1.0	0.0	1.0	1.0	1.0	0.0	1.0
Skier	103	3	1.0	1.0	1.0	1.0	1.0	1.0	1.0
Porcupine	102	6	0.500	0.167	0.333	0.667	0.667	0.500	0.500
Marmot	101	8	0.750	0.500	0.750	0.625	0.875	0.875	0.750
Pika	101	3	1.0	1.0	1.0	1.0	0.667	1.0	1.0
Caribou	94	6	0.833	0.500	0.667	0.500	0.333	0.333	0.500
Indiscernable	87	9	0.889	0.111	0.889	0.889	0.556	0.889	1.0
Hunter	84	4	1.0	0.250	1.0	0.500	0.500	0.750	1.0
Ground Squirrel	79	3	0.333	0.0	0.0	0.333	0.333	0.0	0.0
Badger	75	6	1.0	0.667	0.500	0.667	0.833	0.500	0.833
River Otter	75	2	0.500	0.500	0.0	0.0	0.500	0.500	0.500
Raccoon	67	5	1.0	0.600	0.400	0.600	1.0	0.800	1.0
Snowshoer	65	5	1.0	0.0	0.600	0.600	0.400	0.600	0.800
Common Raven	64	3	1.0	0.333	0.667	0.333	1.0	1.0	1.0
Runner	60	1	0.0	0.0	0.0	0.0	0.0	0.0	0.0
Bald Eagle	58	4	0.750	1.0	0.750	0.500	0.750	0.250	1.0
Beaver	57	3	0.667	0.667	0.667	0.333	0.333	0.667	0.667
Muskrat	57	2	0.0	0.0	0.500	0.500	0.500	0.500	0.500
Cattle	56	3	0.667	0.0	0.667	0.667	1.0	0.667	1.0
Grouse	49	2	1.0	1.0	1.0	1.0	1.0	1.0	1.0
Striped Skunk	48	5	0.600	0.800	1.0	0.600	0.800	0.600	0.800
Fisher	46	1	1.0	0.0	1.0	0.0	0.0	1.0	1.0
Chipmunk	42	4	0.500	0.500	0.500	0.250	0.500	0.500	0.500
Biker	41	3	0.333	0.0	0.333	0.667	0.333	0.667	0.333
Flying Squirrel	38	2	1.0	0.500	1.0	1.0	0.500	0.500	1.0
Mouse	31	1	1.0	1.0	0.0	1.0	0.0	1.0	1.0
Golden Eagle	30	1	1.0	1.0	1.0	1.0	1.0	1.0	1.0
Invertebrate	29	7	1.0	0.429	0.714	1.0	1.0	0.857	1.0
Red Squirrel	25	2	0.500	0.500	0.0	0.0	0.500	0.500	0.500
Small Bird	19	1	0.0	0.0	0.0	0.0	0.0	0.0	0.0
Woodrat	8	2	0.0	0.0	0.0	0.0	0.0	0.0	0.0

### 3.2 Networks used and performance metrics

For this experiment, we compare six modern convolutional neural network architectures: DenseNet201, Inception‐ResNet‐V3, InceptionV3, NASNetMobile, MobileNetV2, and Xception, which collectively represent a spread of architectures that trade‐off computational cost and memory to accuracy. Our experiment also includes all architectures considered as an *ensemble*, where each model votes on a classification in order to produce a final output. Our comparison is in terms of top‐1 accuracy and F1 Score under two conditions: trained locations and untrained locations. *Top‐1 accuracy* considers the percentage of correctly classified species for only the highest confidence prediction. The *F1 score* (also known as the harmonic mean) uses precision and recall to create the F1 score:

$$\text{precision}=\frac{\text{true positives}}{\text{true positives}+\text{false positives}}$$

$$\text{recall}=\frac{\text{true positives}}{\text{true positives}+\text{false negatives}}$$

$$F1=2\times \frac{\text{precision}\times \text{recall}}{\text{precision}+\text{recall}}$$

The F1 score represents the balance of correct predictions for all considered classifications, where low F1 scores occur when a model performs poorly on particular classifications, while overall accuracy may still be high. F1 scores range between 0 and 1 where 0.7 are generally considered well‐performing (Goutte & Gaussier, 2005).

### 3.3 Training augmentation

Due to the limited data and large class imbalance, we used the ImgAug library to incorporate a selection of image augmentation techniques, including mirroring, color channel modifications, blurring, conversion to gray scale, rotation, pixel dropout, pixel cluster normalization, and localized affine transformations for every image sampled (Imgaug github repository‐a). In addition, to improve training time and performance, we used transfer learning to initialize each model with their respective publicly available pretrained weights considering the ImageNet dataset (Krizhevsky et al., 2012). We also supplemented species with very small image numbers by including fixed augmented images as ground truths to reach at least 100 images per classification. These classes can be identified in Table 1 as those with <100 training images.

As an additional step taken to address the classification imbalance, we performed a novel sampling heuristic when selecting training images. For each classification, the ratio of its number of images in comparison with the total number of images of the maximum classification (in our case, this class was the “no animal” class) is calculated. Per epoch, an image is selected at random, followed by a random number generated between 0 and 1. If the random number is greater than the class‐specific ratio, a random sample of the *same class* is selected. Otherwise, if the random number is less than the ratio, a random image from the *entire dataset* is selected and the process repeated. These approaches allow for a stochastic method to include underrepresented classes to the model more frequently in proportion to a particular dataset's class imbalance.

Each model was trained using the adaptive momentum algorithm (ADAM) for 500 epochs using a P100 graphics card utilizing Python 3.6, TensorFlow 2.0, and Keras 2.2 (Kingma & Ba, 2014).

### 3.4 Data protocol for trained locations

For the trained locations experiment, the dataset was split into two parts: one part used for training and the other for testing considering a random split into a 90%–10% ratio of training images to testing images. This was done by generating a random permutation of the range (1, number of images). The first 90% are assigned for training, the rest for testing. Randomizing the distribution of training/testing images is standard practice to ensure no human bias is introduced when selecting a test set (Goodfellow et al., 2016). Images used for the test set are hidden to the model, that is, those images are not included in the training set.

For the trained location condition, images are randomly chosen from all locations using the above process. Table 1 provides the specific number of training versus testing images used per species (left columns). Accuracy results, reported in the following columns of Table 1, are based on running one training/test experiment per network.

### 3.5 Data protocol for untrained locations

Partitioning the dataset for untrained locations followed a somewhat different process using a fivefold cross‐validation split. To explain, *k‐fold cross‐validation* is a standard procedure used in machine learning to assess the performance of machine learning models, especially when they are using novel datasets (Bengio & Grandvalet, 2004; Wong, 2015). Data are partitioned into *k* sets. For each set, that set is used to validate the model, while the remaining sets are used for training.

For our fivefold cross‐validation split, location data were split into two parts, where 80% of the locations were used for training and the remaining 20% of the locations used for testing (this 80/20 split is also standard practice in machine learning). Thus, the training data did not contain any images taken from the test locations. However, this introduced a potential problem. Some species (e.g., Canadian Geese) were only seen at particular locations. If those locations were not included during training, the model would not know about them and would thus exhibit poor performance when attempting to classify images of that species during the test phase. To take this into account, we ran five experiments for each network. While each experiment followed the 80/20 ratio of location splitting, the 20% of locations used for testing varied with each partition. To illustrate with an example, the data are provided with list of locations. We then split this in five distinctly different ways, training each of the six described networks on each of the five splits. The first experiment was trained on images from the first 80% of locations and tested using the latter 20%. The second experiment used the first 0%–60% and last 81%–100% of locations for training, and the middle 61%–80% for testing. The third experiment used 0–40 and 61%–100% for training, and 41%–60% testing. The remaining two followed a similar partitioning pattern. As the five experiments can produce different accuracy results, reports of accuracy will include a ± value highlighting the standard deviation across these tests.

## 4 RESULTS

### 4.1 Model accuracy for trained locations

Table 2 provides the accuracy and F1 scores for the individual networks as well as for the ensemble of networks for the trained location condition. As seen in the accuracy column, each tested network for species classification from camera trap images achieved over 91.0% accuracy. For example, DenseNet201 performed best with an accuracy of 95.6% and an F1 score of 0.794. As a network ensemble, each model votes on a classification, which improved the accuracy and F1 score to 95.9% and 0.812, respectively. The table also reveals that the efficacy of each method is somewhat similar, which suggests other trade‐offs can be considered. For example, MobileNetV2 is designed for efficiency related to size and speed to work on a mobile device and on older PC hardware. It performed competitively with 93.1% accuracy and F1 score of 0.754. For real‐world scenarios with limited PC hardware, this network would be a reasonable choice. If high‐performance computers were available, other networks—including an ensemble—would provide a modest improvement in classification capabilities.

**Table 2:** Summary of performance metrics for Parks Canada species ID with trained locations

Model	Accuracy	F1 score
DenseNet201	**0.956**	**0.794**
Inception‐ResNet‐V2	0.929	0.724
InceptionV3	0.940	0.756
NASNetMobile	0.910	0.714
MobileNetV2	0.931	0.754
Xception	0.954	0.786
Ensemble	**0.959**	**0.812**

Bolded results represent the best performing models.

### 4.2 Model accuracy for new locations

Table 3 provides the accuracy and F1 scores for the individual networks as well as for the ensemble of networks for the untrained location condition. When compared with trained locations as reported in Table 2, we see that the performance of the untrained locations model decreases overall. DenseNet201 once again performed best, with an accuracy of 68.7% and F1 score of 0.698 in Table 2. This is in contrast to its 95.6% accuracy and F1 score of 0.794 in trained locations as reported in Table 1. Considering an ensemble of the models, its performance and F1 score improved to 71.2% and 0.708, respectively (again, underperforming the trained locations ensemble at 95.9% accuracy and 0.812 F1‐Score). While underperforming in comparison with trained locations, these results are significantly better than purely random selection, which would be a 1.81% chance considering the 55 classifications. Interesting of note is that an ensemble improved performance by 2.50% for new locations, while for seen locations it only improved 0.03%.

**Table 3:** Summary of performance metrics for Parks Canada species ID with untrained locations

Model	Accuracy	F1 score
DenseNet201	**0.687** ± **0.057**	**0.698** ± **0.031**
Inception‐ResNet‐V2	0.635 ± 0.049	0.654 ± 0.036
InceptionV3	0.651 ± 0.057	0.655 ± 0.029
NASNetMobile	0.637 ± 0.068	0.678 ± 0.034
MobileNetV2	0.643 ± 0.050	0.653 ± 0.030
Xception	0.685 ± 0.062	0.646 ± 0.034
Ensemble	**0.712**	**0.708**

Bolded results represent the best performing models.

### 4.3 Species recall

Table 1 illustrates the behavior of all the models, as well as an ensemble, considering recall relative to the amount of training data per classification using trained locations. Immediately, two limiting factors become apparent that make it difficult to assess the relationship between training images and classification recall forcing assumptions. First, it is apparent that not all classification tasks are equally difficult for computer vision models. This is indicated when assessing cougars and red foxes, which had a similar number of training images (635 and 688, respectively), where DenseNet201 attained 1.0 and 0.75 recall, respectively. The second is that recall is misleading where there are very few numbers of training/testing image pairs. Table 1 shows the reality of training deep learning models with few numbers of images, where by following a 90%‐10% training/testing split, the number of testing images can become <10, sometimes 1. As a result, recall values of 1.0 appear, which are misrepresentative of recall in practice due to the small sample size.

**Figure 1:**
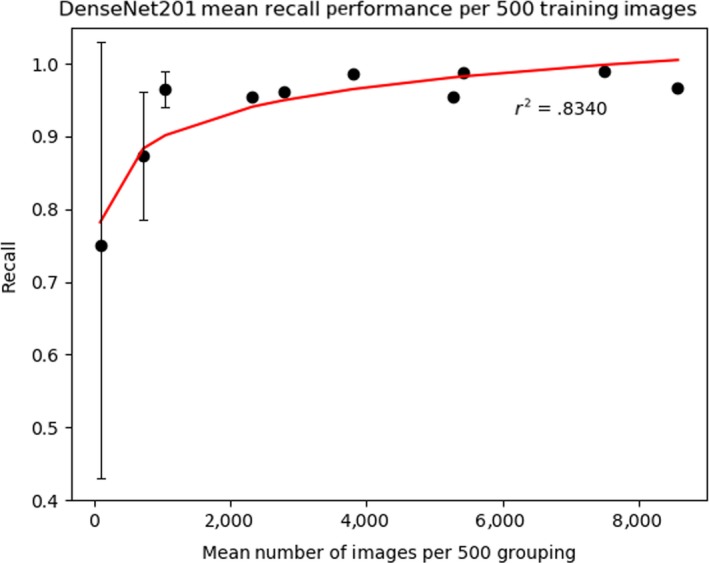
Plot of mean recall considering groupings of 500 training images

Despite these limitations, a relationship of training data to classification recall exists. To account for these discrepancies, we report the correlation between number of training images and recall by taking the mean of groupings per 500 training images for DenseNet201. We correlate this using a logarithmic regression achieving an r2 score of 0.834. Figure 1 graphically visualizes this, where the left side (representing few training images) show highly variable and poor performance that only stabilizes somewhat after approximately 1,000 images. Table 4 details this where images with <500, 500–999, and 1,000+ training images have a mean recall of 0.749 ± 0.329, 0.874 ± 0.103, and 0.971 ± 0.0137, respectively. If one wanted to achieve 95% confidence in their predictions, for our particular dataset, one would need approximately 1,000+ images. These are, of course, rough approximations as the actual recall figure will depend on a variety of factors related to the difficulty of the classification. However, we offer it as a “first‐cut” guideline for ecologists considering current methodologies.

**Table 4:** Comparison of mean and standard deviation of thresholds

Number of images	Number of classifications	Mean
<499 Images	41	0.750 ± 0.329
500–999 Images	6	0.874 ± 0.103
1,000+	8	**0.971** ± **0.0137**

Bolded results represent the best performing models.

## 5 DISCUSSION

### 5.1 What our Results mean to Ecologists

In this paper, we tested the reliability of deep learning methods on a modestly sized and unbalanced ecological camera trap dataset using both trained and untrained location. We set out to test and demonstrate the capabilities of deep learning systems considering a smaller scale ecological dataset and document their success as well as the boundary to which they fail to perform. While previous tests of these deep learning systems have demonstrated very high accuracy levels, their performance is reported on very large amounts of training data (Tabak et al., 2019; Willi et al., 2019; Norouzzadeh et al., 2018). This is problematic. First, large amounts of training data can be impractical to produce for some ecological projects. Second, these accuracy measures can be misleading to those who assuming their limited datasets will behave accordingly. Third, most prior tests also provide little or no report how their models perform on multiple new locations, especially those atypical to those seen during training. Lastly, a single accuracy figure does not give much insight into how accuracy per species can vary given a highly unbalanced datasets. For that purpose, we documented a gradient of performance relative to the amount of training data one has as a guide for ecologists considering deep learning methods for their smaller scale datasets.

Our work offers a series of conclusions. First, our model performs well for smaller scale datasets considering trained locations, with DenseNet201 achieving 95.6% accuracy. This demonstrates that even when using a dataset much smaller than those typically reported within this field, with proper data augmentation techniques and intelligent sampling methods, one can attain high levels of performance. This is very promising for ecological research efforts with limited data where cameras are not relocated over time.

Second, our results show that accuracy is decreased significantly when images used for testing are taken from different camera location sites. To illustrate, DenseNet201 achieved the highest 68.7% accuracy for untrained locations, which is considerably less than the 95.6% accuracy seen for trained locations. This decrease in accuracy likely arises from *overfitting*, where machine learning systems, for example, recognize particular classes only when they appear in combination with certain backgrounds seen during training. While imperfect recognition can still be useful, it does mean that outputs from the model should not be relied upon without human supervision. It also suggests that the high accuracy reported by previous deep learning research in ecology should be considered with some skepticism, especially if they do not include a form of out‐of‐distribution sample testing, of which our k‐fold method is one option, considering different locations as demonstrated here. We recommend that all ecological papers applying deep learning for computer vision standardize to this two‐testing set format. This implies contrasting trained versus k‐fold untrained locations, to ensure their analysis reports a form of out‐of‐distribution locations (Beery, VanHorn, et al., 2019; Tabak et al., 2019).

Third, ecological datasets can be extremely imbalanced. This begs the question: How many training images are required per species when using deep learning methods to attain reasonable performance? We found that species with fewer than training images available (<500) produce highly variable but often poor recall score (Figure 1). This high variance exists, despite our implementation of equivalent sampling, because there are simply are not enough unique examples images of the species for the model to generalize (i.e., make accurate predictions to dissimilar unseen examples). However, as the number of training images available increases, per species recall variability stabilizes and overall performance improves. Stabilizing occurs progressively as additional images are included. Table 1 shows multiple examples of this, such as between Martens (with 223 images, DenseNet201 recall of 0.600) and Coyote (with 893 images, DenseNet201 recall of 0.774). This correlation is not one‐to‐one, however, as not all species classification are equally difficult to distinguish, and few numbered images have very small numbers of testing images skewing the recall metrics. To account for this, we consider the mean recall for 500 image groupings (Table 4). We find that for our dataset, a 1,000+ images per classification are required to achieve consistent performance of 0.95 recall. Of course, further experiments using different datasets would need be run to see whether these figures generalize over other domains that contain similar “messy” camera trap data. While we do not yet know whether these particular figures will apply to other datasets, it does provide a good starting point for further work (Tabak et al., 2019). This answer helps ecologists determine an appropriate cost–benefit threshold, where they could see what the trade‐off is between the number of images needed (which can be costly to acquire) versus the per species recall obtained. For example, if the Parks Canada images were balanced to 1,000 labeled images per 55 classifications, 55,000 training images would be required in total to achieve a predicted >95% recall for all classes.

### 5.2 Contributions and novelty

We expect most ecologists reading this paper will have only passing familiarity with image recognition technology and the prior literature as applied to wildlife camera traps. Because of this, we discuss and highlight the primary contribution of our work in comparison with several recent landmark papers.

To reiterate, the primary focus, contribution, and novelty of our research when compared to prior work is threefold. First, we demonstrate that deep learning is applicable even when only modest‐sized tagged ecological datasets are available for training. Second, we highlight how classification accuracy measures derived from trained locations should not be generalized to predict classification accuracy when images are from multiple new locations. Accuracy will likely degrade significantly. Third, we estimated the lower bounds of images per species required for training to achieve reasonable levels of recognition accuracy. Collectively, our findings can help an ecologist: (a) determine whether deep learning is an applicable approach given their real‐world constraints and (b) determine how many images are required as a whole and per species for deep learning methods to be applied effectively.

Our work is focused on the comparative recognition accuracy of various models when trained on a modest dataset. This is especially important for real‐world domains that do not have large training sets of tagged images available. In contrast, most other prior works use very large training sets, or do not empirically compare different models, or do not explain their choice of model. For example, Tabak et al. (2019) use 3.2 million training images of 27 classes and consider a single model without comparison. While impressive and well performing, Tabak et al. (2019) only test their model's generalization to new locations on a single alternative location considering four species classification and do not document how machine learning systems behave with fewer than thousands of images (their minimum is 1,804 images). To our knowledge, addressing these limiting factors—all key for quantifying and implementing practical deep learning systems for camera traps—has not been done before in a systematic way.

In addition, our methodology is unique, in terms of our combination of data augmentation and novel class sampling. The field of image recognition, especially when applied to camera trap images, is still a young one. While most researchers have developed seemingly similar methodologies for determining accuracy, those methodologies (and thus the results presented by them) actually vary considerably. To help others understand our methodology and to replicate our results with their own datasets, we make our code publicly available as a starting point for others unfamiliar with—or unable to implement their own—deep learning systems for camera trap images (Schneider github animal classification tool).

### 5.3 Practical recommendations using deep learning

Overall, our results demonstrate the successful capabilities of deep learning systems within the ecological domain, albeit with identified limitations. It can be a powerful and practical tool that helps ecologists in the laborious task of extracting ecological information from camera trap images. That being said, the process requires engineering and intelligent foresight when designing and/or using these systems in order for them to perform their best. We discuss our guideline for practical implementation here:

#### 5.3.1 Training data reflect model performance

Machine learning systems generalize to their training data. To further increase performance, one should try to include as many different background locations as possible in your training data. The greater the number, the better the model will generalize (LeCun et al., 2015).

#### 5.3.2 Data augmentation, transfer learning, and classification ratio training

Including a wealth of data augmentation will improve performance by exponentially increasing the number of example images a model sees during training. Transfer learning based on the ImageNet dataset allows for an already trained model to specialize on niche tasks using limited images (Pan & Yang, 2010). Ratio prior training techniques, as performed here, samples training images proportional to their availability in the dataset. This helps improve performance for datasets with high‐class imbalance.

#### 5.3.3 Human‐in‐the‐loop

Before relying on a model, we recommend a human monitor a camera trap system's output and retrain the model as necessary, as outlined in 2019 by Norouzzadeh (Norouzzadeh et al., 2019). Briefly, one monitors the model's output and retrains the model using the example images the model provided incorrect predictions for (we recommend approximately 100). Using this approach, model performance will continually improve, approaching the level of accuracy as the team continues to annotate the images (Holzinger, 2016). Our results show that locations not seen during training decrease performance. As a result, when relocating camera traps, annotating the incorrect model outputs and retraining should greatly improve performance and reliability as that new location will be incorporated in the model (Holzinger, 2016).

#### 5.3.4 Object detection

While not previously discussed here in detail, object detection networks, such as Faster R‐CNN, are trained to localize classifications within an image rather than classify the image as a whole (Ren, He, Girshick, & Sun, 2017). Training a generic “animal” object classifier can be used to extract the pixel representation of animals from images, which can then be passed through a separate species classifier for a niche tasks. This approach has multiple advantages including the ability to count the number of animals in an image, as well as decrease the noise present from the backgrounds of images, and has been demonstrated previously as a viable technique (Schneider et al., 2018).

### 5.4 Future work

The realm of possibilities for future work combining rapidly advancing deep learning methods with camera trap imagery is vast. We offer a mere few suggestions here.

First, we recognize that our results are reported on a single dataset. Thus, future work should repeat this experiment with other datasets, considering trained and untrained locations, in order to see how accuracy varies between them and to determine what can be generalized. Performing k‐fold validation on Snapshot Serengeti and other citizen scientist datasets of decreasing size would provide a gradient as to how many background locations are required for generalization to new locations (Willi et al., 2019).

Second, camera trap images differ from typical machine learning task as they are not independent of one another. Each camera captures many images over time from the same static location with the same field of view. For example, cameras will often capture an animal or herd of animals as they move through the scene as a series of related images. Thus, one can take advantage of temporal components to improve the recognition accuracy of animal individuals as they appear. In 2019, Beery, Wu, et al. (2019)) explore this, and we believe there is continued room for exploration in this domain.

Third, the ability to generalize to new background locations is a result of a model overfitting to particular background images. Data augmentation could mitigate overfitting. For example, we could use a *semantic segmenation network*, such as MASK‐RCNN, that is trained to extract the pixels of the animals seen in a camera trap image (He et al., 2017). Using this, one could then perform data augmentation by pasting cropped animal images onto millions of backgrounds, allowing the model to become agnostic to backgrounds, and thus able to generalize to any unseen location.

Fourth, the human‐in‐the‐loop must complement deep learning. Deep learning classifications are not 100% accurate. Errors will creep in as false positives and false negatives. An analyst should be able to rapidly and efficiently review images and their classifications, and correct them as needed. Software designed specifically for this purpose can help. One emerging example is the Timelapse image analysis system, which facilitates image and classification review. It draws bounding boxes on all detected entities where its intensity varies with its confidence, and it lets the analyst filter images by a particular classification (e.g., people). The analyst can then review all images falling under that classification via rapid replay, thus detecting incorrect classifications reasonably quickly and reliably (Greenberg, Godin, & Whittington, 2019).

Fifth, one area where we believe machine learning and large scale ecological datasets have not yet merged is *semi‐supervised learning* (Zhu & Goldberg, 2009). Having excess amounts of data, but limited numbers of labels, is a common occurrence for camera trap data projects. Semi‐supervised learning has the ability to leverage both labeled and unlabeled data when training on classification tasks. The use of semi‐supervised learning for camera trap images is an area of untapped potential.

Sixth, we focus largely on domain shift, demonstrated here as the inability of machine learning systems to generalize to new locations when trained on camera trap data. There is an entire area of machine learning focused on accomplishing this task, known as *domain adaptation* (Csurka, 2017). One such method is known as domain adversarial training. This approach involves training a network to answer the animal classification correctly while answering the background incorrectly. This forces the model to ignore information relative to the background and will improve generalization when considering new locations. This could be a promising way to address the camera trap domain shift problem.

Lastly, we believe the future of deep learning models will improve beyond species reidentification to individual reidentification. Human re‐ID is nearly a solved problem, and preliminary work has been performed on primates and humpback whales using deep learning relying on a library of training data for each individual (Deb et al., 2018; Humpback whale identification challenge; Taigman, Yang, Ranzato, & Wolf, 2014). Deep learning approaches for similarity comparison do not require example images of every individual within a population and show promise for animal reidentification considering multiple species (Schneider, Taylor, & Kremer, 2020). If a deep learning model could provide reliable animal reidentification from camera traps, one could perform autonomous population estimation of a given habitat using a mark and recapture sampling technique (Robson & Regier, 1964). If applied to real‐world camera trap data successfully, such a system could be used to model a variety of ecological metrics, such as diversity, relative abundance distribution, and carrying capacity, all contributing to larger overarching ecological interpretations of trophic interactions and population dynamics.

## 6 CONCLUSION

Recent advancements in the field of computer vision and deep learning have given rise to reliable methods of image classification, with caveats. We demonstrate the successful training of six deep learning classifiers capable of labeling 55 species and human activities from 36 unique geographic locations trained from a modest number of difficult ecological camera trap image data. For all models, we saw above 91.0% accuracy in trained locations and 65.5% accuracy for untrained locations. We found DenseNet201 performed best with 95.6% and 68.7% accuracy for seen and unseen locations, respectively. We find that when using trained locations, classifications with <500 images had low and highly variable recall of 0.750 ± 0.329, while classifications with over 1,000 images had a high and stable recall of 0.971 ± 0.0137. As a result, we offer as a guideline ecologists have at least 1,000+ labeled images per species classification of interest as a training standard when working with camera trap data in order to achieve 0.95 species classification recall. To ensure ecologists can compare our findings with their own datasets, we make our code publicly available, where it is designed for any ecologist to use. Our findings show promising steps toward the automation of the laborious task of labeling camera trap images, which can be used to improve our understanding of the population dynamics of ecosystems across the planet.

## CONFLICT OF INTEREST

None declared.

## AUTHOR CONTRIBUTIONS

Stefan Schneider is a PhD Candidate in the department of Computational Science and was the primary author for the work responsible for the written content, structure, papers selected, figure, and tables. Saul Greenberg is a professor who collaborates with Parks Canada and other agencies to develop software for camera trap image management, including TimeLapse (Greenberg et al., 2019). Dr. Greenberg was responsible for providing the camera trap images and editing the document with an ecological audience in mind. Graham Taylor who is an associate professor of Engineering and a member of the Vector Institute for Artificial Intelligence provided feedback on its presentation, content, and best practices for the machine learning methods. Stefan C. Kremer, who is a professor of Computer Science specializing in machine learning and bioinformatics, provided editorial comments on both structure and content as well as advice and guidance throughout the candidate's studies.

## Data Availability

The data for this experiment are from Parks Canada, contains human faces and are protected under a nondisclosure agreement. To ensure ecologists can conduct similar experiments with other datasets, we have open sourced our code and made it publicly available (Schneider github animal classification tool).
